# Predictability of food supply modulates nocturnal hypothermia in a small passerine

**DOI:** 10.1098/rsbl.2020.0133

**Published:** 2020-06-03

**Authors:** Johan F. Nilsson, Jan-Åke Nilsson, Juli Broggi, Hannah Watson

**Affiliations:** Evolutionary Ecology, Lund University, SE-223 62, Sweden

**Keywords:** thermoregulation, body temperature, hypothermia, food supply, energetics, birds

## Abstract

The combination of short days and long cold winter nights, in temperate regions, presents a major challenge for small diurnal birds. Small birds regularly employ heterothermy and enter rest-phase hypothermia during winter nights to conserve energy. However, we know little about how environmental conditions, such as food availability, shape these strategies. We experimentally manipulated food availability in winter to free-living great tits *Parus major*. A ‘predictable' and constant food supply was provided to birds in one area of a forest, while birds in another area did not have access to a reliable supplementary food source. We found that predictability of food affected the extent of nocturnal hypothermia, but the response differed between the sexes. Whereas male nocturnal body temperature was similar regardless of food availability, females exposed to a naturally ‘unpredictable' food supply entered deeper hypothermia at night, compared with females that had access to predictable food and compared with males in both treatment groups. We suggest that this response is likely a consequence of dominance, and subdominant females subject to unpredictable food resources cannot maintain sufficient energy intake, resulting in a higher demand for energy conservation at night.

## 1. Introduction

Energy is the currency of life, and animals must obtain sufficient resources to meet their metabolic demands. Challenging environmental conditions, such as those experienced during winter in cold temperate regions, may place constraints on an organism's ability to acquire sufficient energy resources for survival [1]. Winter in cold regions is especially challenging for small, diurnal birds. Small birds demand high energy intake to fuel a high metabolic rate, in part resulting from a high rate of heat loss owing to a large surface-area-to-volume-ratio [2–4]. This becomes especially critical during the nocturnal roosting period, when individuals require sufficient energy resources to survive the long winter night, but a combination of low food availability, low ambient temperatures (*T*_a_), inclement weather and short days can impose energetic constraints.

Small passerine birds can reduce their body temperature (*T*_b_) and enter rest-phase hypothermia to conserve energy reserves while roosting at mid to high latitudes [5,6]. By reducing the temperature gradient between the body and external air, hypothermia can significantly reduce heat loss and energy expenditure. The use of nocturnal hypothermia by small diurnal birds could reduce metabolic demands by as much as 50% [7] and increase winter survival by up to 58% [8]. However, birds do not consistently use a heterothermic strategy, suggesting that a regulated reduction in *T*_b_ carries costs, such as increased predation risk [9,10], altered sleep patterns [11] and reduced efficiency of cellular processes and immune function [12]. It is therefore important to understand both the determinants and costs of nocturnal hypothermia in birds.

The extent of hypothermia is strongly associated with *T*_a_, hormone levels and breeding cycle [5,6]. Studies in captivity suggest that the ability to obtain sufficient energy reserves is critical in the control of the extent of nocturnal hypothermia. Green pigeons (*Treron calvus*) exposed to restricted food in aviaries reduced nocturnal *T*_b_ to a greater extent than those fed *ad libitum* [13], and fasting induced deeper nocturnal hypothermia in domestic pigeons (*Columba livia domestica*) [14]. Similarly, when exposed to food *ad libitum* in outside aviaries, wintering blue tits (*Cyanistes caeruleus*) did not enter nocturnal hypothermia, which contrasted with those in the wild that did reduce *T*_b_ at night [15]. While food availability has been shown to influence thermoregulatory strategies of birds, our limited knowledge comes from captive studies and, to the best of our knowledge, no study has experimentally tested the effect of food supply on nocturnal *T*_b_ regulation in the wild (but see [16]). In the present study, we manipulated predictability of food supply in the wild throughout winter and quantified the effects on nocturnal hypothermia in free-living great tits *Parus major*. We predicted that great tits with access to a predictable food supply would maintain a higher *T*_b_ at night, compared with birds with a naturally unpredictable food supply, owing to expected variation in the ability to acquire energy resources.

## 2. Material and methods

The study was carried out in a continuous tract of mixed coniferous/deciduous forest in southern Sweden (55°39′07.7″N, 13°34′14.0″E). Great tits use nest-boxes for nocturnal roosting in winter, facilitating capture of birds during winter nights. Eleven feeding stations, spaced 200–380 m apart, were regularly maintained, throughout October 2016–March 2017, to provide a *predictable* and constant supply of food (approx. 50:50 sunflower seeds:peanuts) to birds. From here on, we refer to this as the ‘predictable' treatment area. The average distance between a nest-box and a feeder in the predictable area was 0.12 ± 0.07 km (mean ± s.d.). Another area within the same forest received no food supplementation, and thus birds wintering in this area were reliant on natural food resources, which are typically *unpredictable* in winter and certainly less predictable than permanent feeding stations. From here-on, we refer to this as the ‘unpredictable' treatment area. Nest-boxes in the unpredictable area were situated within 1.37 ± 0.27 km (mean ± s.d.) from a feeder in the predictable area. This distance is considerably larger than the typical winter home range of a great tit (0.05 km^2^ [17]). Neither abiotic nor biotic factors are likely to vary markedly between the areas. There were no significant differences in *T*_a_ between the predictable (mean ± s.e.: 1.09 ± 0.06°C) and unpredictable (mean ± s.e.: 1.05 ± 0.06°C) areas during the study period (*p* = 0.31; measured with iButtons (DS 1922 L, Maxim integrated, USA) attached to nest-boxes).

Between 1 January and 12 February 2017, 82 (predictable = 44; unpredictable = 38) great tits were captured while roosting in nest-boxes at night. A temperature-sensitive passive integrated transponder (BioTherm13, Biomark, USA) was implanted subcutaneously in the neck. Sex and biometrics (body mass and wing length) were recorded, and all birds were marked with a uniquely numbered metal ring. Birds were aged as either juvenile (in their first winter, 1) or adult (in their second winter or older, 2+). Following the procedure, birds were returned to the nest-box, and the entire handling time was less than 10 min. Between 13 February and 13 March, 152 ‘snapshot' measurements of *T*_b_ (i.e. a single measurement per individual per night) were collected from 57 tagged individuals (predictable = 26; unpredictable = 31), while roosting at night, using a portable radio frequency identification reader (Biomark HDR Plus with racket antenna). By briefly holding the antenna close to the base of a nest-box, identity and current *T*_b_ of a roosting bird were instantaneously recorded, without disturbance. Measurements were collected between 18:00 and 23:20. No birds were ‘recaptured' outside the treatment area in which they were originally captured and marked.

### (a) Statistical analyses

For each measurement of *T*_b_, we derived numerous variables relating to *T*_a_: mean, minimum and maximum relating to (i) present night, (ii) present day, (iii) previous 24 h, (iv) previous night, (v) previous day, (vi) previous 72 h and (vii) previous 168 h. Day and night periods were classified as 0700–1700 and 1700–0700, respectively, and 24 h periods as 0700 to 0700. Linear mixed models with normal error structures were fitted to data on *T*_b_ using lmerTest in R 3.2.4 [18]. A saturated model included the two-level factors of treatment (predictable/unpredictable), sex (female/male) and age (1/2+), the covariates of body mass, wing length, date and time, and the interaction between treatment and sex. A random effect of individual identity was included. We first independently tested each of the variables relating to *T*_a_ to identify the variable that explained most variation in *T*_b_. Model selection then proceeded using the full model above plus minimum *T*_a_ from the previous 72 h; terms were eliminated if *p* > 0.1 when comparing a reduced model (dropping one term at a time) to the original model in a likelihood ratio test. Significance levels were estimated using conditional *F*-tests based on Satterthwaite approximation for the denominator degrees of freedom. Post-hoc tests were carried out using difflsmeans().

## 3. Results

**Figure 1.**
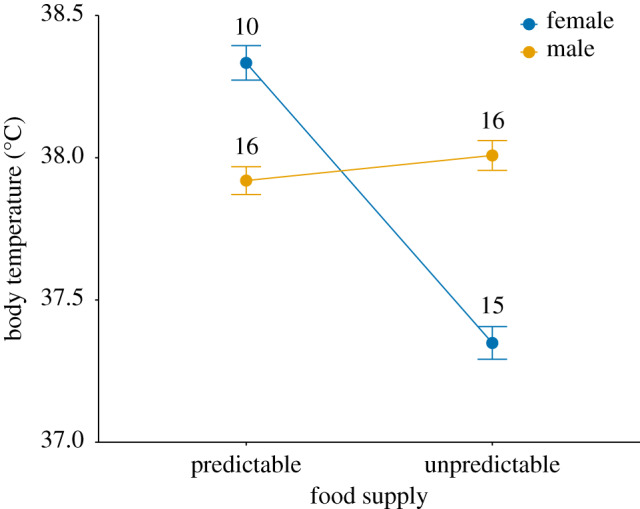
Nocturnal *T*_b_ of wild female and male great tits exposed to a *predictable* and *unpredictable* supply of food in winter. Fitted means ± s.e. from a minimum adequate linear mixed model controlling for age, time, *T*_a_ and individual identity. Numbers indicate sample sizes.

A significant interaction between treatment and sex revealed that *T*_b_ responses to the level of food predictability differed between males and females (figure 1; treatment:sex: *β*_unpredictable:male_ = 0.957 ± 0.319, *F*_1,45.2_ = 8.97, *p* = 0.004). Post-hoc tests confirmed that males showed a similar nocturnal *T*_b_ when exposed to either a predictable or unpredictable food supply (*p* = 0.9); by contrast, females exposed to an unpredictable food supply exhibited a lower *T*_b_ compared with females exposed to a predictable food supply (*p* < 0.001) and compared with males in both treatment groups (*p* = 0.009). Adult birds displayed lower *T*_b_ than juvenile birds (*β*_adult_ = −0.361 ± 0.162, *F*_1,48.8_ = 4.93, *p* = 0.03). There was a tendency for *T*_b_ to be lower later in the evening (*β* = −0.114 ± 0.0614, *F*_1,129.7_ = 3.42, *p* = 0.07) and when mean *T*_a_ during the previous 72 h was lower (*β* = 0.0361 ± 0.0193, *F*_1,134.6_ = 3.48, *p* = 0.06). There was no significant effect of date, body mass or wing length (all *p* > 0.6) on *T*_b_.

## 4. Discussion

To the best of our knowledge, this is the first study to quantify nocturnal thermoregulatory activities of birds in response to a manipulation of food availability in the wild (but see [16]). While food deprivation has been shown to induce reductions in nocturnal *T*_b_ in birds, studies have been performed in captivity and imposed a more extreme scenario of reduced food availability [13,14]. Employing a similar manipulation of food availability in the wild, Cornelius Ruhs *et al*. [16] found no effect of winter food supply on the change in *T*_b_ following an immune challenge, but they did not report the nocturnal hypothermic response. We demonstrated that females exposed to an unpredictable food supply entered deeper nocturnal hypothermia, compared with males in both treatments and females that have access to a predictable and constant supply of food at nearby feeding stations. Conversely, males maintained the same nocturnal *T*_b_, regardless of food availability.

If birds are exposed to a limited food supply, a reduction in *T*_b_ at night is expected to reduce energy demands, improve conservation of energy resources and, subsequently, increase the chances of survival to the following morning [8]. Despite this expectation, male great tits were able to maintain the same thermoregulatory strategy independent of food supply. While dominance is unlikely to directly affect thermoregulatory capacity [19], dominant individuals are likely to have priority at food resources even when they are naturally unpredictable. It has been shown that adult male great tits have priority access to food and consequently higher predictability of foraging success in winter, which means that they do not increase winter fat reserves as much as subordinates––juveniles and females [20]. Even if natural food supply is not as predictable as that provided by a permanent feeding station, our results suggest that the ability of males to dominate access to natural food resources enables them to maintain sufficient energy intake to avoid the need to enter deeper hypothermia at night. By contrast, subordinate females exposed to a limited food supply are likely to experience lower foraging success and need to achieve greater reductions in nocturnal *T*_b_ to offset the increased risk of over-night starvation. Conversely, juvenile birds, which are also subordinate, demonstrate a consistently higher *T*_b_ than older birds, irrespective of the predictability of food supply. A similar age-related difference was shown by Andreasson *et al*. [10] and could reflect a lack of experience in young birds, making them deploy a sub-optimal strategy. Indeed, since this is the first winter experienced by juvenile birds, their strategy of maintaining a higher *T*_b_ has not been proven successful, and selective disappearance of birds demonstrating this strategy could create the apparent age-related variation. However, we did not see any evidence for selective disappearance of juveniles over the course of the experiment.

When exposed to an environmental temperature of 0°C, the mountain chickadee (*Poecile gambeli*)––a closely related species to the great tit––can reduce its energy expenditure by approximately 12% by entering nocturnal hypothermia [7]. According to the equation *M* = *C*'(*T*_b_ – *T*_a_) (where *M* = metabolic rate and *C*' = thermal conductance, e.g. [21]), females in the unpredictable group would have experienced an energy saving of approximately 2.7% per night at the temperatures to which they were exposed in this study. Over the course of the winter, this would likely amount to a considerable energetic benefit. Despite the large energy savings of nocturnal hypothermia, not all of our individuals reduced their *T*_b_ to the same extent, providing evidence for a cost–benefit trade-off. Theoretical and empirical evidence suggests that predation risk has a strong effect on a bird's decision to enter nocturnal hypothermia [10,22]. Even in a state of rather shallow hypothermia, great tits are unable to detect predator scent [23].

Another potential cost associated with nocturnal hypothermia is a reduced capacity or efficiency of cellular repair and maintenance systems, possibly coupled with elevated release of free radicals (though there is evidence for both increases and decreases in production of reactive oxygen species in hypothermic animals [24,25]). Research has so far focused on hibernating mammals, which undergo large and extended reductions in *T*_b_. Hibernating mammals show elevated levels of lipid peroxides [26], yet commonly also have increased levels of antioxidants [27,28]. However, a study on rats showed that an acute cold exposure can increase lipid peroxidation and reduce antioxidant enzymes *in vivo* [24]. Although altered redox homeostasis appears to be common among hibernating mammals, the underlying mechanisms and consequences of such changes are likely to be very different, compared with the smaller daily modulations in *T*_b_ exhibited by small birds.

Irrespective of the potential costs of nocturnal hypothermia, this experimental study clearly demonstrates the importance of energy availability and foraging constraints in regulating nocturnal hypothermia in small birds in winter. Undergoing large reductions in nocturnal *T*_b_ can provide an insurance strategy when energy resources are limited. Entering hypothermia is likely to be adaptive, even if it incurs ecological and physiological costs. Understanding the underlying costs of short-term hypothermia is fundamental to fully understand the nature of the trade-offs governing nocturnal hypothermia, and future studies should seek to obtain a holistic understanding of how small wintering birds manage their energy budgets in relation to food availability.

## Data accessibility

Data available from the Dryad Digital Repository: https://doi.org/10.5061/dryad.dbrv15dxp [29].
